# Using AI-predicted protein structures as a reference to predict loss-of-function activity in tumor suppressor breast cancer genes

**DOI:** 10.1016/j.csbj.2024.10.008

**Published:** 2024-10-05

**Authors:** Rohan Gnanaolivu, Steven N. Hart

**Affiliations:** a Department of Quantitative Health Sciences, Mayo Clinic, Rochester, MN, United States; b Department of Laboratory Medicine and Pathology, Mayo Clinic, Rochester, MN, United States

**Keywords:** AlphaFold2, FoldX, ESMFold, Protein stability, Free energy, BRCA1, AlphaMissense, Breast cancer

## Abstract

**Background:**

The loss-of-function (LOF) classification of most missense variants in tumor suppressor breast cancer genes *BRCA1, BRCA2, PALB2*, and *RAD51C* remains unclassified and confounds clinical actionability. Classifying these variants is challenging due to their rarity, leading clinicians to rely on *in silico* predictive methods. Protein stability changes are associated with function, making stability predictors valuable. Stability predictions upon missense variant perturbations require high-resolution protein structures. However, the availability of these high-resolution structures is lacking. This study explores using generative AI to predict high-resolution protein structures, which can then be analyzed with *in silico* protein stability prediction methods to assess LOF activity in ordered regions of the protein. This study also determines the appropriate *in silico* protein stability and dedicated *in silico* missense prediction methods in dbNSFP v4.7 database to predict LOF activity in ordered regions of these four genes. Functional classifications from homology recombination DNA repair (HDR) assays and variant classifications from the ClinVar database provide a reliable dataset for evaluating the performance of these *in silico* prediction methods.

**Results:**

Complex AlphaFold2 structures of the BRCA1-C terminal (BRCT) domain and the DNA-binding (DB) domain of *BRCA2,* analyzed using protein stability tool FoldX predicts LOF activity from missense variants significantly better than experimentally-derived structures in ordered regions. The BRCT domain achieved an Area Under the Curve (AUC)= 0.861 (95 % CI:0.858–0.863) and AUC= 0.842 (95 % CI:0.840–0.845), while the DB domain achieved an AUC= 0.836 (95 % CI:0.8322–0.841), compared to AUC= 0.847 (95 % CI:0.844–0.850) and AUC= 0.835 (95 % CI:0.832–0.837) from the BRCT domain, and AUC= 0.830 (95 % CI:0.821–0.8320) from the DB domain from experimentally-derived structures. Protein stability does not predict LOF activity from missense variants better than dedicated *in silico* missense predictors. Overall, we find that AlphaMissense ranks highly, with an average AUC= 0.890 (95 % CI 0.886–0.895) from ordered regions across these four cancer genes, compared to all other *in silico* missense predictors present in the dbNSFP database.

**Conclusions:**

The study reveals that generative AI protein predicted structures can outperform experimentally-derived structures in evaluating LOF activity from predicted protein stability in ordered regions of genes BRCA1, BRCA2, PALB2 and RAD51C. The study also highlights the predictive performance of AlphaMissense as the premier *in silico* missense prediction method to predict LOF activity from missense variants in these four tumor suppressor breast cancer genes. The code for this study can be downloaded for free on GitHub (https://github.com/rohandavidg/CarePred)

## Graphical Abstract

**Figure:**
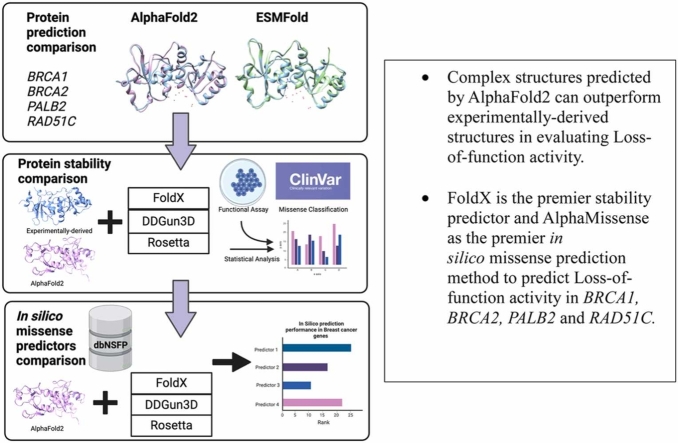

## 1 Background

Tumor suppressor genes *BRCA1, BRCA2, PALB2*, and *RAD51C* play crucial roles in homology-directed recombination DNA repair (HDR) activity, and mutations in these genes have been implicated in breast cancer [1]. While the functional impact of truncating mutations in these genes are well characterized, the clinical impact of > 95 % of all possible missense mutation in *BRCA2, PALB2* and *RAD51C* and > 80 % of all possible missense mutations in *BRCA1* remain unclassified, therefore, most are commonly referred to as variants of uncertain significance [2]. Various *in silico* and in vitro functional assays have been employed to evaluate functional activity. Commonly used *in silico* methods primarily use features such as sequence conversation, protein conformation and stereochemical properties of the amino acid as features to infer functional outcomes [3], [4], [5]. Researchers and Clinician have also utilized *in silico* stability predictors, which predict the destabilization or over-stabilization of the protein to infer loss or gain of function. Consequently, the absolute value of predicted ΔΔG has been used to evaluate protein function [6], [7], [8].

Calculating protein stability involves measuring the effect and change in free energy of the protein based on the presence of a mutation. The change in protein structure directly impacts overall stability, particularly in haploinsufficient genes like *BRCA1, BRCA2, PALB2*, and *RAD51C* [9], [10], [11], [12]. One method to assess protein stability is by estimating the difference in free energy of unfolding of the proteins (ΔG) between the wild-type and variant protein: ΔΔG = ΔG_variant – ΔG_wildtype. FoldX [13], Rosetta [14], and DDGun3D [15] are well-known stability predictors that induce a mutation from a given wild-type protein structure and predict ΔΔG. Recent publications have demonstrated that FoldX, Rosetta and DDGun3D had the highest spearman rank correlation coefficient (rho) with functional measurements from deep mutational scanning data utilizing experimentally-derived protein complexes from the protein data bank (PDB) compared to other stability predictors[16]. However, the impact of stability on function is protein dependent, as there are several factors such as sequence composition, post-translation modification, haploinsufficiency, and binding partners that influence stability of the protein [17], [18]. The functional assays available for genes BRCA1, BRCA2, RAD51C and PALB2, measure the loss-of-function (LOF) activity from a missense variant, and the predictive performance of these protein stability methods to predict LOF in these tumor suppressor breast cancer genes is still relatively unknown. The study of protein function using predicted protein stability involves several limitations as well, such as the inherent variability of these methods [19] and the availability of experimentally-derived crystalized structures of the complete protein.

Advances in generative AI, particularly in protein prediction models like AlphaFold2 (AF2) [20] and ESMFold [21], have shown promise in predicting structures that are highly similar to structures found in the PDB. AF2 and ESMFold were shown to predict protein structures that are highly similar to experimentally-derived structures in the Critical Assessment of Structure Prediction (CASP)15 challenge [22]. Recent CASP13 [23] and CASP14 [24] challenges highlighted the giant leap in advancement in protein structure prediction, with results highlighting the accuracy of predicted protein structures. Recent studies have demonstrated that features extracted from AF2 structures are effective in predicting the functional classification of missense variants [25]. However, the ability of using generative AI protein prediction methods in HDR pathway genes to predict protein stability is relatively unknown, with recent publications underscoring the reliability of protein stability predictions from structures generated by homology-based tools, particularly when sequence identity is at least 40 %, highlighting the need for accurate wild-type structures in the prediction of protein stability from a missense variant [26], [27].

Experimental methods, such as multiplexed assay of variant effect (MAVE) and functional assays provide insight on LOF activity from missense variants in *BRCA1, BRCA2, RAD51C*, and *PALB2* [28], [29], [30], [31], [32], [33]. MAVE assays in these genes measure the HDR activity based on survival or growth of the cells upon the induced mutations. Results from these assays provide a reliable set of mutations to evaluate protein stability on the functional activity in our genes of interest [34]. However, these MAVE assays are shown to have high stochasticity, and hence multiple replicates are often required. MAVE assays are also limited based on the biological mechanism involved and do not provide mutational classification on all possible missense mutations in these genes, with some assays being domain specific to the gene of interest.

*In silico* missense predictors are considered the weakest form of evidence to classify a missense variant [5]. REVEL [35] and BayesDel [36] are two *in silico* missense prediction tools that have been historically cited to accurately predict deleteriousness in genes predisposed to breast cancer [37]. Newer, deep learning models such as AlphaMissense [38] and MetaRNN [39] have gained attention in predicting LOF activity. The effectiveness of these newer predictors, as well as those predicting protein stability based on functional classifications from MAVE experiments in these genes, is still largely unexplored.

The goal of this study was to assess how protein stability impacts LOF activity from missense variants in BRCA1, BRCA2, PALB2 and RAD51C. We used predicted protein structures from generative AI tools as the baseline wild-type structural templates, which were analyzed with protein stability methods FoldX, Rosetta, and DDGun3D to evaluate functional outcomes. Based on the Area Under the Curve (AUC), Our results demonstrate that FoldX, Rosetta, and DDGun3D can predict LOF from missense variants using AF2 wild-type structures, with performance comparable to the prediction derived from experimentally-derived structures found in the PDB. Furthermore, AF2 wild-type structures enhance LOF predictions analyzed with FoldX in the BRCA1-C terminal (BRCT) domain of *BRCA1* and the DNA-Binding (DB) domain in BRCA2, compared with the experimentally-derived structures. FoldX was also shown to significantly outperform Rosetta and DDGun3D to predict HDR activity from the MAVE assays in the BRCT domain of *BRCA1* and the DB domain in *BRCA2*. Finally, *in silico* predictor AlphaMissense ranked as the top predictor to predict LOF activity based on the AUC in the ordered regions of *BRCA1* and *BRCA2*, as well as it was found to be one of the top predictors in *PALB2* and *RAD51C*.

## 2 Methods

### 2.1 Data Selection

The dataset used to evaluate the LOF activity was a combination of ClinVar missense variants listed in dbNSFP v4.7 [40] and the LOF classifications from missense variants derived from MAVE assays for genes BRCA1, BRCA2, PALB2, and RAD51C (Additional file 1: Table S1). The MAVE assay for *BRCA1* was based on saturation Genome editing in HAP1 cell lines that had a total of 2086 mutations over the N-terminal RING and BRCT functional domains that aim to measure the LOF activity from missense variants in this cell line [29]. For *BRCA2*, we used the functional classification from a HDR cell-based assay from 462 missense variants affecting the *BRCA2* DNA binding domain [30]. For *RAD51C*, we used the functional classification from a HDR reporter assay, which introduced 174 missense variants in mammalian hRAD51C expression constructs using site-directed mutagenesis [31]. For *PALB2*, we used the functional classifications from 91 missense variants evaluated from a HDR assay from two separate studies [32], [33].

We further supplemented our dataset with the mutational classification from the ClinVar database listed in dbNSFP 4.7 release. We group all classification of pathogenic and likely pathogenic into the “functionally damaging” category, and classification of benign and Likely benign into the “functionally neutral” category.

### 2.2 PDB selection

We chose eleven structures from the PDB (Additional file 1: Table S2), that represent different ordered regions of genes *BRCA1, BRCA2, RAD51C* and *PALB2*. We chose these structures based on their high resolution and coverage. These structures exist as a subunit and as a complex, with two or more protein chains representing a complex or as a single chain representing a subunit. For *BRCA1*, we downloaded 4OFB[41], 1JNX[42], 1T15[43] and 7LYB[44], as these structures represent the two ordered domains of *BRCA1* (BRCT and RING). 1JNX is a single chain subunit structure, 1T15 and 4OFB are complex structures that contains 2 protein chains each, representing the BRCT domain of *BRCA1*. 7LYB is a 7-protein chain complex structure, where chain ‘M′ represents the RING domain of *BRCA1*. For *BRCA2*, we downloaded 1MJE[45], 1IYJ[45] and 1MIU[45] which are two-protein chain complex structures and represents the ordered regions of the DB domain of *BRCA2*. Chain A in 1MJE and 1MIU represents the DB domain of BRCA2, whereas chain B in 1IYJ represents the DB of BRCA2. For *RAD51C*, we downloaded structures 8FAZ[46] and 8OUZ[47], which are complexes with 4 protein chains, In 8FAZ, chain “C” represents the entire gene of RAD51C, whereas chain B represents entire gene of RAD51C. For *PALB2*, we chose the 3EU7[48] structure, a complex with two chains, with chain “A” representing the entire *PALB2* gene. Additionally, we downloaded the 2W18[48] structure, a subunit representing the *PALB2* gene.

### 2.3 AlphaFold2 and ESMFold prediction

AF2 structures for eleven high-resolution PDB structures were generated using ColabFold v1.5.5 [49] using the pdb100 templates. Default settings were applied for all remaining configurations (Additional file 1: Table S3). To generate these structures, the FASTA sequence from each experimentally-derived structure was extracted using BioPython [50], and subsequently used as inputs to generate five separate replicates, which were then ranked based on model confidence. The PDB entries 4OFB, 1JNX, 1T15, 7LYB, IMJE, 1IYJ, 1MIU, 8FAZ, 8OUZ, 3EU7, and 2W18 were selected for structure prediction. AF2's multimer prediction mode was employed, with all eleven structures being predicted based on homology templates, and no de-novo structures were generated.

Additionally, we used the python implementation of ESMFold to generate structures for nine out of the eleven experimentally-derived structures using their FASTA sequences as input. For these predictions, we employed ESM-2 pretrained model “esm2_t33_650M_UR50D,” which comprises 650 million parameters and 33 layers, running on a single T4 GPU. For each structure, we generated five replicates. Due to ESMFold’s limitation of generating structures with a maximum of 1024 amino acids, complete structures could not be generated for complex 7LYB and 1IYJ.

### 2.4 Protein stability

FoldX, Rosetta and DDGun3D predictors were used to predict ΔΔG utilizing both AF2 and experimentally-derived structures as wild-type templates for all possible missense mutations, leading to a total of 246,910 mutations. Using the python module pyFoldX [51], which uses FoldX 5.0 the structures were passed through the “RepairPDB '' function prior to ΔΔG calculations, with default settings. For every mutation, five replicates were computed and the mean ΔΔG value was calculated. DDGun3D python package was used to generate predicted ΔΔG using the default settings, five replicates were computed and the mean ΔΔG value was calculated. The Cartesian ΔΔG application from Rosetta suite (Linux build 2021.16.61629) was used to generate the ΔΔG predictions, following standard protocols published in several publications [52], [53] while using the *Ref2015* scoring function. The structures were initially relaxed, and ΔΔG predictions were generated over three iterations. The results were averaged to produce the mean ΔΔG values in (Rosetta energy per unit). To convert the units into kcal/mole scale, a scaling factor of 2.94 was applied, as shown in the literature [16], [52].

### 2.5 Statistical analysis

A Mann-Whitney U statistical test was employed from the SciPy [54] stats python package to understand the association of predicted |ΔΔG| generated from FoldX, Rosetta and DDGun3D to protein LOF activity. The metric Root Mean Square Deviation (RMSD) calculations were computed using the *superimposer* method from the BioPython v1.79 PDB package. RMSD was used as the metric to evaluate the similarity between the AF2 structure compared to the experimentally-derived structure downloaded from the PDB. Similarity was calculated using BioPython v1.79 PDB package over the entire structure and over the carbon-alpha (Cα) backbone chain. We use SciPy v1.7.3 stats package to compute the rho between predicted |ΔΔG| from AF2 structures and experimentally-derived structures analyzed with FoldX, Rosetta and DDGun3d. We also compute the rho of |ΔΔG| from AF2 structures and experimentally-derived structures analyzed with FoldX, Rosetta and DDGun3d, compared to the continuous functional MAVE activity score. To compute the confidence interval of the rho, we applied a Fischer transformation to the correlation coefficients. Utilizing these Fisher transformations, we calculated the combined standard errors and the z-scores of the difference. The p-values were then derived from the z-scores based on the normal distribution. No corrections were applied for multiple testing.

To calculate the predictive ability of the *in silico* missense predictors and stability predictors to predict LOF activity from missense variants from ordered regions in BRCA1, BRCA2, PALB2 and RAD51C, we used the metric AUC from the *roc_auc_score* method from the scikit-learn v1.0.2 [55] package. We computed the AUC, the AUC under the precision-recall curve and the false positive rates, along with their 95 % confidence intervals. This was achieved by sampling 200 times across the dataset while balancing out the class labels. The python code used for this study is available at https://github.com/rohandavidg/CarePred.

## 3 Results

### 3.1 Comparison of generative AI protein prediction structures

From the literature, two protein structures are considered similar if the RMSD between carbon-alpha (Cα) back-bone chains that are superimposed is < 3.8 Å [56]. Using this threshold as the metric for similarity, AF2 generates 3D protein structures comparatively similar to the experimentally-derived structures compared to ESMFold from the 9 structures analyzed representing the domains in BRCA1, BRCA2, PALB2 and RAD51C. RMSD of < 3.8 Å threshold was generated for only 3 structures (4OFB, 1JNX and 2W18) by ESMFold, and due to the size limitation of 1024 residues, 2 complex structures (7LYB, 1IYJ) were not analyzed. AF2 predicts 9 structures less than the RMSD threshold, with on 1MIU and 1IYJ failing this threshold (Table 1). 1MIU and 1IYJ could not be predicted without significant errors and hence they were excluded from further analyses. Among the 9 structures with a Cα back-bone RMSD of < 3.8 Å, the per-residue distances was calculated by superimposing the predicted AF2 structures onto the experimentally-derived counterparts. This analysis revealed that over > 98 % of the residues in 8 out of 9 structures had a distance < 3.8 Å. The mean distance for these 8 structures ranged from 1.32 Å (1T15) to 2.31 Å (1MJE), highlighting the prediction accuracy of AF2. However, one structure, 7LYB had a mean residue distance of 19 Å, even though the distances between the *BRCA1* Cα back-bone chain and its experimentally-derived counterpart was < 3.8 Å (Additional File 1: Fig. 1).

**Table 1:** Root Mean Square Deviation (RMSD) values of AlphaFold2 and ESMFold structures superimposed on experimentally-derived structures from the PDB.

**GENE**	**PDB ID**	**Residue Count**	**AF2 subunit backbone (RMSD) Å**	**ESMFold Subunit backbone (RMSD) Å**	
*RAD51C*	8FAZ	926	1.04		7.06
*RAD51C*	8OUZ	924	1.77		7.02
*BRCA1*	7LYB	1389	1.16		NA
*BRCA1*	4OFB	223	0.4		1.53
*BRCA1*	1T15	219	0.51		1.43
*BRCA1*	1JNX	207	0.8		1
*BRCA2*	1IYJ	1272	9.28		NA
*BRCA2*	1MIU	713	6.36		8.90
*BRCA2*	1MJE	648	2		7.46
*PALB2*	3EU7	327	0.66		9.79
*PALB2*	2W18	306	1.08		1.36

**Fig. 1:**
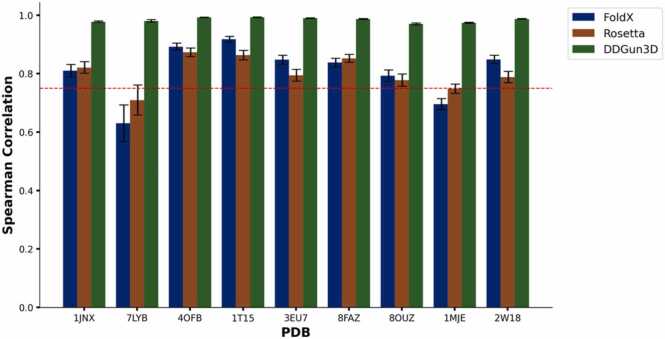
Spearman rank correlation coefficient denoting the correlation between the predicted |ΔΔG| derived from experimentally-derived structures vs AlphaFold2 structures as the wild-type template analyzed with protein stability predictors FoldX, Rosetta and DDGun3D. The red line at y = 0.75 indicates the threshold for strong correlation.

### 3.2 Association of protein function from predicted |ΔΔG| generated from experimentally derived structures

The Mann-Whitney U statistical test was used to assess the association of |ΔΔG| values derived from nine experimentally derived structures (PDB ID:1JNX, 4OFB, 1T15, 7LYB, 1JME, 3EU7, 2W18, 8FAZ and 8OUZ), representing ordered regions of *BRCA1, BRCA2, PALB2* and *RAD51C*. Protein stability predictions were generated using FoldX, Rosetta and DDGun3D, and tested against the functionally damaging classification from missense variants in the ClinVar database and the LOF classification from MAVE assays. Our analysis revealed a significant association of predicted |ΔΔG| and LOF activity in all four genes (Additional file 1: Fig. 2).

**Fig. 2:**
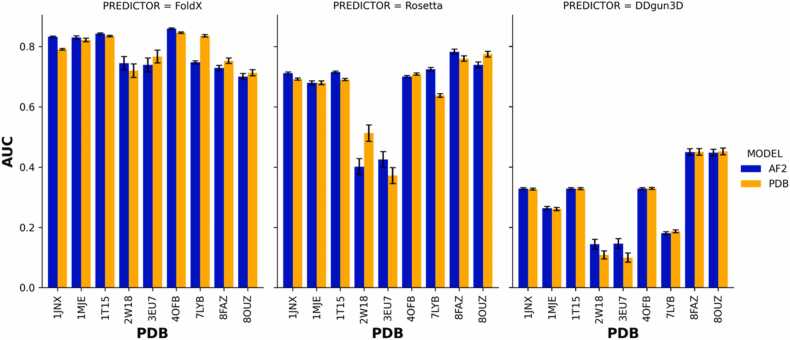
Area under curve denoting the predictive ability of |ΔΔG| from experimentally-derived structures vs AlphaFold2 structures as the wild-type template to predict loss-of-function activity in BRCA1, BRCA2, PALB2 and RAD51C analyzed with protein stability predictors FoldX, Rosetta and DDGun3D.

### 3.3 Linearity of |ΔΔG| generated from protein stability predictions using AF2 structures compared to experimentally-derived structures

With only 3 ESMFold structures meeting the Cα backbone threshold < 3.8 Å, and higher RMSD values compared to AF2 structures, all subsequent analyses excluded ESMFold structure predictions. Using the nine AF2 structures that had an RMSD of the Cα backbone < 3.8 Å, we calculated the rho between |ΔΔG| values predicted from AF2 structures and experimentally-derived structures analyzed with FoldX, Rosetta and DDGun3D. Our analysis revealed *a* strong correlation in |ΔΔG| predictions across FoldX, Rosetta, and DDGun3D using both AF2 structures and experimentally-derived structures (Fig. 1). FoldX and Rosetta had a rho value > 0.75 in 7 out of the 9 structures, indicating strong correlations, with 7LYB and IMJE being the only exception. FoldX generated a rho value of 0.63 (95 % Confidence Interval (CI) 0.60–0.66) with 7LYB and 0.70 (95 % CI 0.690.70) with 1MJE. Rosetta had a rho value of 0.71 (95 % CI 0.68–0.73) with 7LYB and 0.75 with 1MJE (95 % CI: 0.74–0.75). DDGun3D showed a strong correlation for all 9 structures, with a mean rho value > 0.97.

To assess whether the heterogeneity of the rho value was impacted by the features from AF2 structures, we tested the monotonic relationship of the |ΔΔG| differences between the predicted |ΔΔG| derived from AF2 structures and experimentally-derived structures, analyzed with FoldX, Rosetta and DDGun3D with the features from AF2 structures. A rho value was calculated, and no monotonic relationship was observed between the |ΔΔG| differences and the AF2 features (Additional file 1: Fig. 3A). We further examined whether the heterogeneity of the |ΔΔG| differences was dependent on the per-residue distance (Å) between the superimposed AF2 structure and the experimentally-derived structure. The results showed that the rho was not significantly impacted by per-residue distances for up to 4 Å (Additional file 1: Fig. 3B). However, there was a positional effect on heterogeneity, with certain amino acids at specific positions contributing disproportionately to the variability in the prediction from FoldX and Rosetta.

**Fig. 3:**
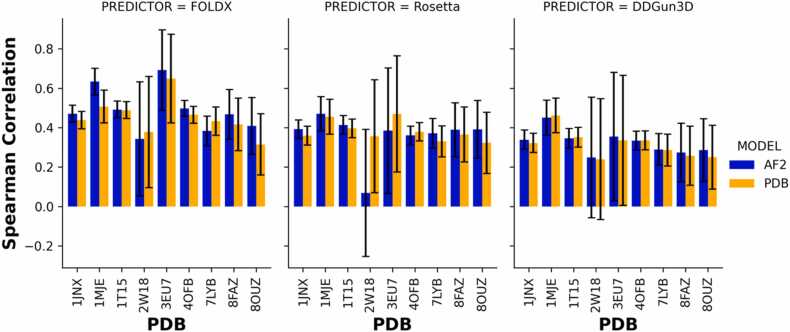
Spearman rank correlation coefficient denoting the correlation of predicted |ΔΔG| from experimentally-derived structures vs AlphaFold2 structures with the continuous measurement of functional HDR activity in genes *BRCA1, BRCA2, PALB2* and *RAD51C* utilizing the |ΔΔG| predictions from protein stability predictors FoldX, Rosetta and DDGun3D.

### 3.4 Comparison of predicted |ΔΔG| from experimentally-derived structures vs AF2 structures to predict LOF

The predicted |ΔΔG| from AF2 structures analyzed using FoldX were significantly at predicting LOF activity from missense variants than the experimentally-derived structures analyzed with FoldX in the BRCT domain of *BRCA1* and the DB domain in BRCA2 (Fig. 2). In the BRCT domain of *BRCA1*, FoldX generated an AUC= 0.861 (95 % CI:0.858–0.863) and AUC= 0.842 (95 % CI:0.840–0.845) from the predicted AF2 complex structures of 4OFB and 1T15 respectively, compared to AUC= 0.847 (95 % CI: 0.844–0.850) and AUC= 0.835 (95 % CI:0.832–0.837) from the experimentally-derived complex structures of 4OFB and 1T15. Similarly, FoldX generated an AUC= 0.836 (95 % CI:0.833–0.839) from the AF2 subunit structure of 1JNX, compared to an AUC= 0.792 (95 % CI:0.789–0.795) from the experimentally-derived subunit structure of 1JNX. Upon caparisons of the AUC’s, Our results indicate that complex AF2 structure enhances the prediction of LOF activity in the BRCT domain of *BRCA1*. However, in the RING domain of *BRCA1*, experimentally-derived complex structure of 7LYB analyzed using FoldX was significantly better than the AF2 complex structure of 7LYB, with an AUC= 0.835 (95 % CI=0.831–0.840), compared to AUC= 0.741 (95 % CI:0.735–0.748). The AF2 complex structure of 7LYB was noted to have a mean per residue distance of 19 Å when superimposed on the experimentally-derived structure, which was outlier compared to the other 8 structures that had a mean per residue distance of < 3 Å, indicating structural difference from the side-chains.

In *BRCA2*, FoldX predictions of LOF activity from missense variants were significantly better than Rosetta and DDGun3D, and sub-setting to the mutations to the DB domain of BRA2, AF2 complex structure of 1MJE analyzed using FoldX generated an AUC= 0.8364 (95 % CI:0.8322–0.8407), which was significantly better than an AUC= 0.8299 (95 % CI:0.8213–0.8320) that was generated with the experimental-derived complex structure of 1MJE analyzed using FoldX.

In *PALB2*, there was no significant difference between the predicted |ΔΔG| from the AF2 complex structure and the experimentally-derived complex structure analyzed using FoldX. Among the stability predictors, FoldX predictions of LOF activity from missense variants were significantly better than Rosetta and DDGun3D. Analyzing the AF2 complex structure of 3EU7, FoldX generated an AUC= 0.721 (95 % CI:0.694–0.747), compared to Rosetta and DDGun3D, which generated an AUC= 0.401 (95 % CI:0.375–0.427) and AUC= 0.157 (95 % CI:0.135–0.179) respectively. With the AF2 subunit structure of 2W18, FoldX generated an AUC= 0.719 (95 % CI: 0.695–0.743), which was significantly better than Rosetta and DDgun3D, with an AUC= 0.359 (95 % 0.329–0.388) and AUC= 0.159 (95 % CI:0.138–0.181) respectively.

In *RAD51C*, we find that Rosetta predictions of LOF activity were significantly better than FoldX and DDGun3D. The Rosetta prediction of |ΔΔG| analyzed using the AF2 complex structure of 8FAZ generated an AUC= 0.782 (95 % CI:0.773–0.791) which was significantly better than AUC= 0.760 (95 % CI 0.751–0.769) generated from the experimentally-derived structure of 8FAZ. However, with structure 8OUZ, Rosetta analyzed with the experimentally-derived complex of 8OUZ generated an AUC= 0.774 (95 % CI:0.765–0.781), was significantly better than AUC= 0.739 (95 % CI:0.730–0.748) that was generated from with the AF2 structure of 8OUZ.

We evaluated the prediction of |ΔΔG| values from the experimentally-derived structures and AF2 structures using rho against the continuous HDR functional activity measurements from MAVE assays. We found no significant difference in the correlation between functional activity and |ΔΔG| between the nine AF2 structures or its experimentally derived counterparts, analyzed with FoldX, Rosetta or DDGun3D (Fig. 3). However, there was heterogeneity from the prediction among the stability predictors. The rho values derived from the |ΔΔG| predictions using the AF2 complex structure of 4OFB (representing *BRCA1* BRCT domain) and 1MJE (representing BRCA2-DSS1-ssDNA complex), as analyzed with FoldX, were significantly higher compared to those from AF2 structures or the experimentally-derived structures analyzed with Rosetta and DDGun3D. For 4OFB, the complex AF2 structure analyzed with FoldX resulted in a rho= 0.497 (95 % CI:0.455–0.537), compared to rho= 0.360 (95 % CI:0.312–0.407) from the AF2 structure analyzed by Rosetta, and rho= 0.334 (95 % CI:0.285–0.381) from the AF2 structure analyzed by DDGun3D. Similarly, for the 1MJE AF2 structure, a rho= 0.634 (95 % CI:0.562–0.696) was observed, compared to rho= 0.470 (95 % CI:0.378–0.553) from the AF2 structure analyzed by Rosetta, and rho= 0.470 (95 % CI:0.357–0.535) from the AF2 structure analyzed by DDGun3D. In *PALB2*, and *RAD51C*, there was no significant difference between FoldX, Rosetta and DDGun3D to predict the continuous HDR functional activity.

### 3.5 Comparison of protein stability predictors with *in silico* missense predictors

**Fig. 4:**
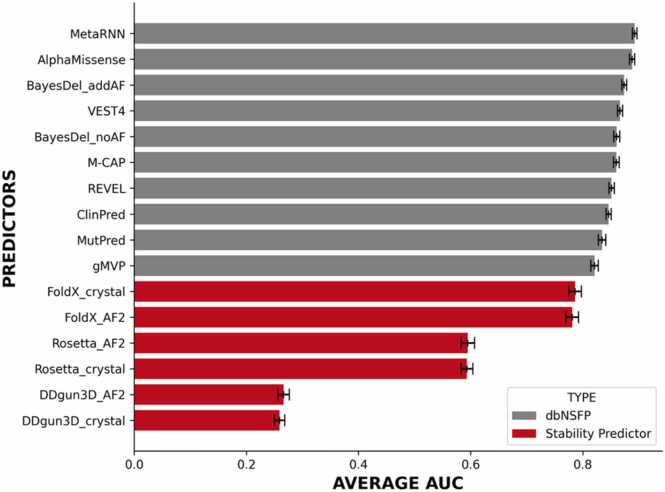
Area under the curve of the top 10 *in silico* missense predictors vs stability predictors to predict loss-of-function activity in *BRCA1, BRCA2, RAD51C* and *PALB2* stratified by in silico missense predictors found in the dbNSFP database (in grey) vs protein stability predictors (in red).

We find that existing *in silico* missense predictors significantly outperform protein stability predictors in predicting LOF activity caused by missense variants, based on the area under the curve (AUC) (Fig. 4). Additionally, no single *in silico* missense predictor emerges as a clear choice for predicting LOF activity in all four genes based on the AUC. When considering the average AUC from all four genes, we find that MetaRNN and AlphaMissense are the leading predictors, with MetaRNN achieving an average AUC= 0.895 (95 % CI:0.891–0.902) and AlphaMissense reaching an average AUC= 0.890 (95 % CI 0.886–0.896) (Additional file 2). To determine the most appropriate *in silico* missense or stability predictor for predicting LOF activity in ordered regions across all four genes, we rank ordered the predictions for all predictors in the dbNSFP v4.7 database and stability predictors, calculating the average rank across all four genes based on the AUC.

**Fig. 5:**
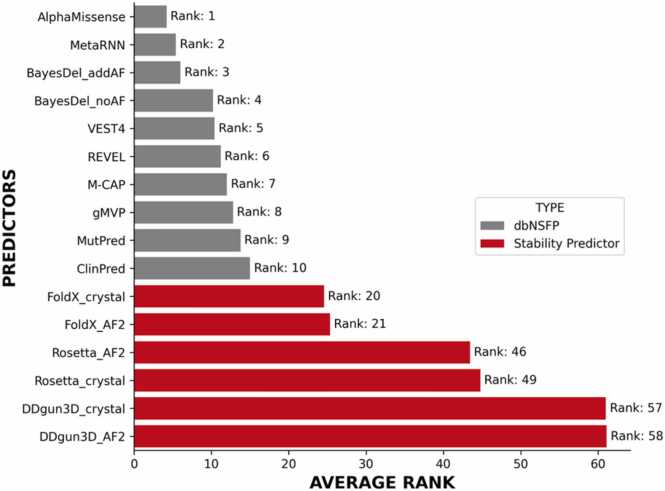
Rank ordered performance of the top 10 *in silico* missense predictors vs stability predictors to predict loss-of-function activity in *BRCA1, BRCA2, RAD51C* and *PALB2* stratified by *in silico* missense predictors found in the dbNSFP database (in grey) vs protein stability predictors (in red).

From the rank ordered results (Fig. 5), we find that AlphaMissense had the highest average rank, followed by MetaRNN, and BayesDel_addAF. Among the stability predictors, FoldX, using AF2 wildtype structures as the wild-type template, achieved a rank of 21. This is including the |ΔΔG| predictions from AF2 complex 7LYB to predict LOF activity. This indicates that predicted |ΔΔG| from FoldX outperforms 32 other predictors in the dbNSFP database for predicting LOF activity. However, we should note that the mutations used for this evaluation are enriched with mutations that are in ordered regions of the proteins.

We also evaluated the false positive rates (FPR) for all dbNSFP predictors and stability predictors. Our analysis showed that AlphaMissense, MetaRNN, and gMVP had the lowest FPRs, with values comparable to one another (Additional file 1: Fig. 5). Specifically, AlphaMissense had an average FPR of 0.139 (95 % CI: 0.130–0.148), MetaRNN had an average FPR of 0.143 (95 % CI: 0.134–0.152), and gMVP had an average FPR of 0.146 (95 % CI: 0.137–0.156) across the ordered regions for all four genes.

## 4 Discussion

### 4.1 AF2 predictions compared to ESMFold

AF2 and ESMFold are considered two well-known protein structural predictors and consistent with existing literature, AF2 multimer predicts protein structures that are highly similar to experimentally-derived structures compared to ESMFold in terms of the RMSD in *BRCA1, BRCA2, PALB*2, and *RAD51C*. ESMFold predictions are comparatively faster than AF2, but the predictions are limited to 1024 amino acids, thereby larger structures, such as 7LYB and 1IYJ were not compared. AF2 is more suited for this study for the high similarity and its ability to predict large protein structures. Two AF2-predicted structures, 1IYJ and 1MIU, which represents the BRCT domain of BRCA2, exhibited an RMSD exceeding the 3.8 Å Cα backbone threshold. The discrepancy in 1IYJ is likely due to nearly 50 % of the protein being predicted with a sequence identity of less than 50 %, thereby yielding a structure with areas of low PLDDT. With 1MIU, certain regions across the structure resulted in low sequence coverage and the predicted aligned error revealed regions of high error across regions that are representative of the DB domain.

### 4.2 Association of predicted protein stability to protein Loss-of-function

Consistent with existing literature, we found that there is a strong association of stability towards LOF activity using all three protein stability predictors, suggesting that protein destabilization or over-stabilization is an important factor in the contribution to LOF activity from missense variants in ordered regions of *BRCA1, BRCA2, PALB2* and *RAD51C* [7], [8]*.* These genes have been reported to be haploinsufficient, suggesting that minor perturbations to the protein could result in destabilization, which could impact function and lead to damaging effects. We also chose FoldX, Rosetta and DDGun3D as these predictors demonstrated strong performance in terms of rho results with deep mutational scan data using complex experimentally-derived protein structures, and also these predictors require a wildtype protein template to generate its predictions [16].

### 4.3 Prediction from DDGun3D is less dependent on wild-type protein templates

Overall, strong rho was observed between the |ΔΔG| prediction using either AF2 structure of the experimentally-derived structure, with the exception for 7LYB and 1MJE. We find that DDGun3D predictions showed stronger correlations compared to FoldX and Rosetta. This could be due to the fact that only 33 % of DDGun3D predictions is based on the wildtype protein structure, while the remaining contribution comes from Blosum62 matrix substitution scores, difference in statistical potential score from the linear chain of the amino acid between the wildtype and mutant, and the difference between the hydrophobicity of the wild-type and mutant [15]. Whereas, FoldX and Rosetta use a mixture of physics and statistical methods to compute protein stability based on the clashes caused by introducing a mutant within the wild-type structures.

### 4.4 Protein structural features have no impact on protein stability predictions

Recent publications have highlighted the use of protein structural features from AF2 structures to study LOF activity [25]. Features such as Predicted Local Distance Difference Test (PLDDT), Relative Solvent Accessibility (RSA), distance between residues, physicochemical properties such as atomic weight, isoelectric points and aromaticity were shown to be useful in predicting LOF activity. We tested the dependence of the difference in |ΔΔG| prediction from experimentally-derived structure and AF2 structures with these features. Our results showed no influence on the difference in stability prediction to the difference in features values, thus suggesting that these features are independent of protein stability and hence have independent effects on the relationship with LOF from missense variants in BRCA1, BRCA2, PALB2 and RAD51C. Thereby we can make a claim that stability, along with features such as PLDDT, RSA, distance between residue, physicochemical properties such as atomic weight, isoelectric points and aromaticity can be used to study protein LOF from missense variants in these four genes.

### 4.5 FoldX prediction using AF2 wild type structures predicts loss-of-function better than other stability predictors

Overall, our results support the choice of using FoldX as the preferred protein stability predictor for these genes, utilizing complex AF2 structures as the wild-type input, compared to Rosetta and DDGun3D. Notably, our findings suggest that analyzing AF2 structures, which are highly similar to experimentally-derived structures (<4 Å Cα backbone and side chains) with FoldX predicts LOF activity just as good as prediction from complex experimentally-derived structures analyzed with FoldX. Our results also suggest that there is a domain specific effect on the predictiveness of stability towards function in *BRCA1*, suggesting that the degree of perturbation is heterogeneous across domains. Even though the predictions are based on several replicates, we acknowledge that there is inherent noise in the predictions from FoldX, Rosetta and DDGun3d. These predictors were not inherently built to predict protein LOF activity from missense variants, but experimental protein stability.

### 4.6 AlphaMissense predictions in tumor suppressor breast cancer genes

Analysis based on the AUC metric revealed that *In silico* missense predictors predict functional consequences better than protein stability predictors in *BRCA1, BRCA2, RAD51C* and *PALB2*. AlphaMissense consistently ranks highly in the prediction of LOF activity from missense variants in ordered regions in all four genes, and on average AlphaMissense emerges as one of the top-ranking predictors. BayesDel is currently considered the *in silico* model of preference by the ClinGen Variant Curation Expert Panel [30].

(https://cspec.genome.network/cspec/ui/svi/doc/GN092), however newer predictors, such as, AlphaMissense and MetaRNN predict LOF activity significantly better than BayesDel in the ordered regions of these four breast cancer genes based on the AUC metric. AlphaMissense utilizes a transformer based multiple sequence alignment of protein sequences, whereas MetaRNN is an ensemble neural network model that uses many existing *in silico* missense predictors as features and hence there is an argument that the predictions might be overfit to the training data to the individual features.

In summary, Generative AI tools such as AF2 multimer predictions can be used in the prediction of protein stability in haploinsufficient genes *BRCA1, BRCA2, PALB2* and *RAD51C* to study LOF activity from a missense variant. However, these predictions are primarily focused on mutations in ordered regions and do not account for disordered regions of the protein complex. The application of generative AI to study functional consequence in disordered regions was beyond the scope of this study. While we acknowledge the limitations of ClinVar data, particularly the circularity issue where training data used to develop *in silico* missense predictors may be used in their evaluation, this concern is especially pronounced in ensemble based methods employed in the development of these *in silico* missense predictors [57]. Additionally, MAVE assays provide an independent dataset for evaluating LOF activity from missense variants, though they are not without limitations. These assays are known to have errors due to the type of assay used, experimental artifacts, and variability in replicates. While these assays will not replace computational methods in the near future, they contribute to refining our understanding of the factors causing damaging effects on protein function.

## Ethics approval and consent to participate

Not applicable.

## Funding

Not applicable.

## CRediT authorship contribution statement

**Steven N. Hart:** Writing – review & editing, Supervision, Project administration, Conceptualization. **Rohan Gnanaolivu:** Writing – original draft, Methodology, Formal analysis, Data curation.

## Declaration of Competing Interest

The authors declare that they have no known competing financial interests or personal relationships that could have appeared to influence the work reported in this paper.
