# Supplementary material for: Using AI-predicted protein structures as a reference to predict loss-of-function activity in tumor suppressor breast cancer genes
Source: Comput Struct Biotechnol J. 2024 Oct 5;23:3472–80. doi: 10.1016/j.csbj.2024.10.008 (PMC11490748; doi:10.1016/j.csbj.2024.10.008)
Supplement: Supplementary file 1 — Supplementary material [file mmc1.docx]

## Additional file: Supplemental

**Table S1: Table denoting the total number of classified mutations used from the ClinVar database and MAVE functional assays for evaluation.**

| **GENES** | **Functional** | | | **ClinVar** | |
| --- | --- | --- | --- | --- | --- |
|  | **Neutral** | **Deleterious** | **Intermediate** | **Neutral** | **Deleterious** |
| *BRCA1* | 1476 | 441 | 169 | 720 | 324 |
| *BRCA2* | 313 | 137 | 12 | 336 | 935 |
| *PALB2* | 84 | 7 | 0 | 31 | 271 |
| *RAD51C* | 137 | 30 | 7 | 3 | 57 |

**Table S2: List of PDB ID from Genes used in this study.**

| **GENE** | **PDB ID** | **Atom count** | **Resolution** | **Residue count** | **Protein chains** |
| --- | --- | --- | --- | --- | --- |
| *RAD51C* | 8FAZ | 7331 | 2.30 Å | 926 | 4 |
| *RAD51C* | 8OUZ | 7449 | 2.20 Å | 924 | 4 |
| *BRCA1* | 7LYB | 14699 | 3.28 Å | 1389 | 7 |
| *BRCA1* | 4OFB | 1791 | 3.05 Å | 223 | 2 |
| *BRCA1* | 1T15 | 1906 | 1.85 Å | 219 | 2 |
| *BRCA1* | 1JNX | 1660 | 2.50 Å | 207 | 1 |
| *BRCA2* | 1IYJ | 10092 | 3.40 Å | 1272 | 2 |
| *BRCA2* | 1MJE | 5198 | 3.50 Å | 648 | 2 |
| *PALB2* | 3EU7 | 2585 | 2.20 Å | 327 | 2 |
| *PALB2* | 2W18 | 2543 | 1.90 Å | 306 | 1 |

**Table S3: Parameters used in ColabFold and ESMFold**

| **ColabFold parameters** | |
| --- | --- |
| template mode | pdb100 |
| model_type (complex) | alphafold2_multimer_v3 |
| model_type (monomer) | alphafold2_ptm |
| num_cycles | 3 |
| recycle_early_stop_tolerance | 0.5 |
| relax_max_iterations | 200 |
| pairing_strategy | greedy |
|  |  |
| **ESMFold Parameter** | |
| pretrained model | esm2_t33_650M_UR50D |
| repr_layers | 33 |
| chunk size | 128 |
| num_recycles | 4 |


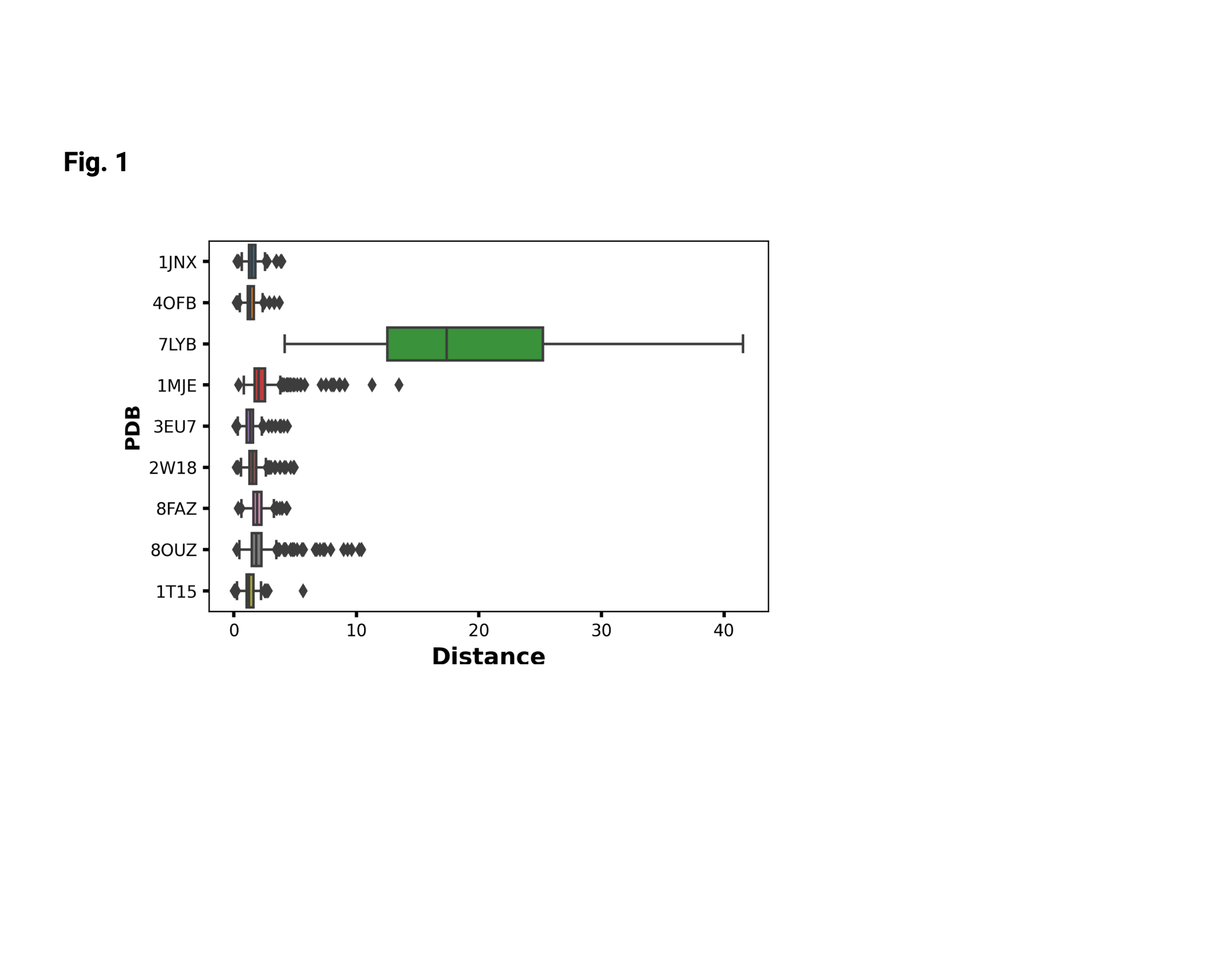


Figure 1: Distribution of the per residue distance in Å between AlphaFold2 predicted structure and experimentally-derived structure found in the PDB. AlphaFold2 prediction of 7LYB was an outlier with a mean residue distance of 19Å


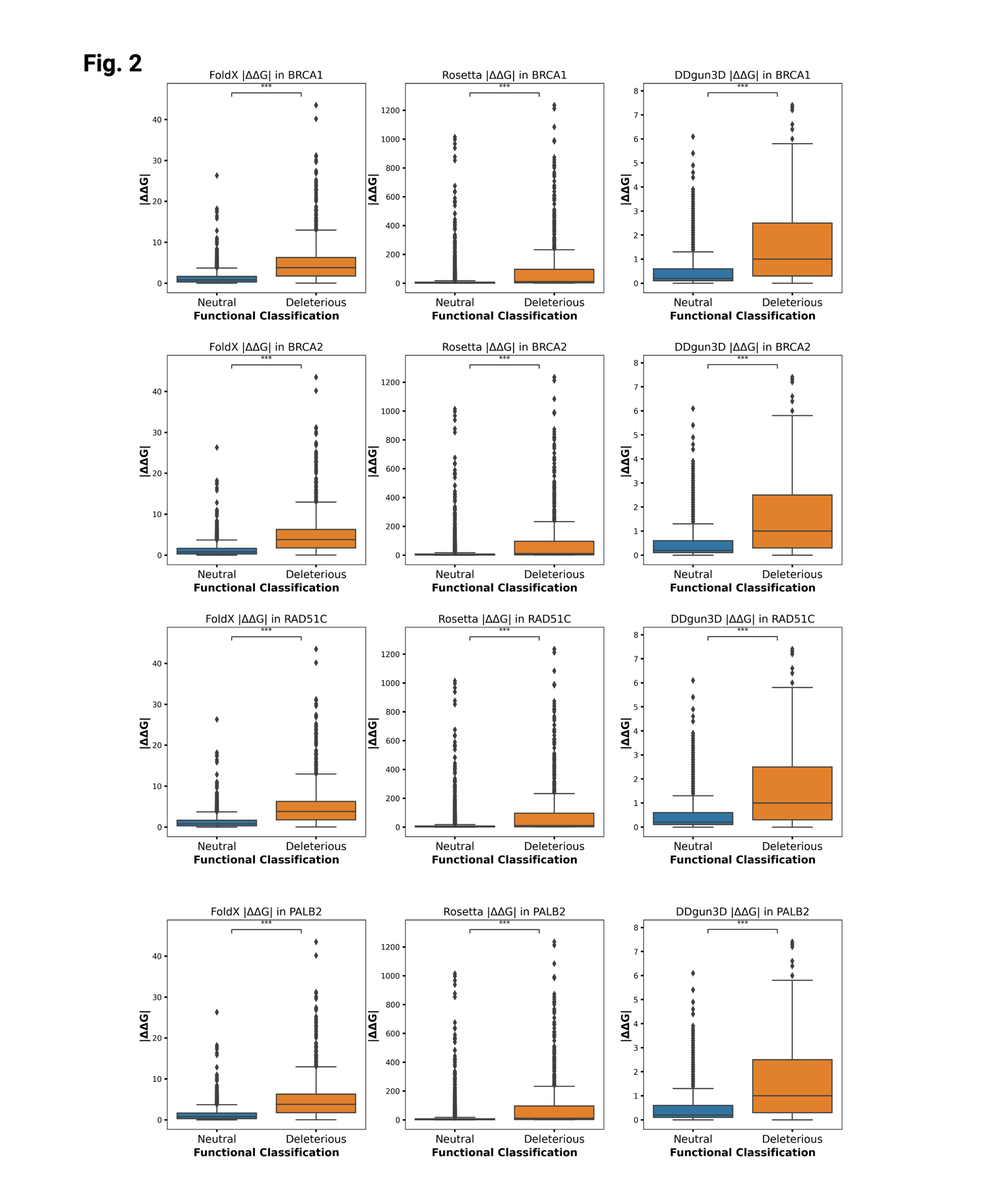


Figure 2: Distribution of predicted |ΔΔG| from experimentally-derived structure analyzed with FoldX, Rosetta and DDGun3D stratified by functional classification (Deleterious vs Neutral) in genes *BRCA1, BRCA2, PALB2* and *RAD51C*, with the association between the two groups denoted by the Mann-Whitney U test.


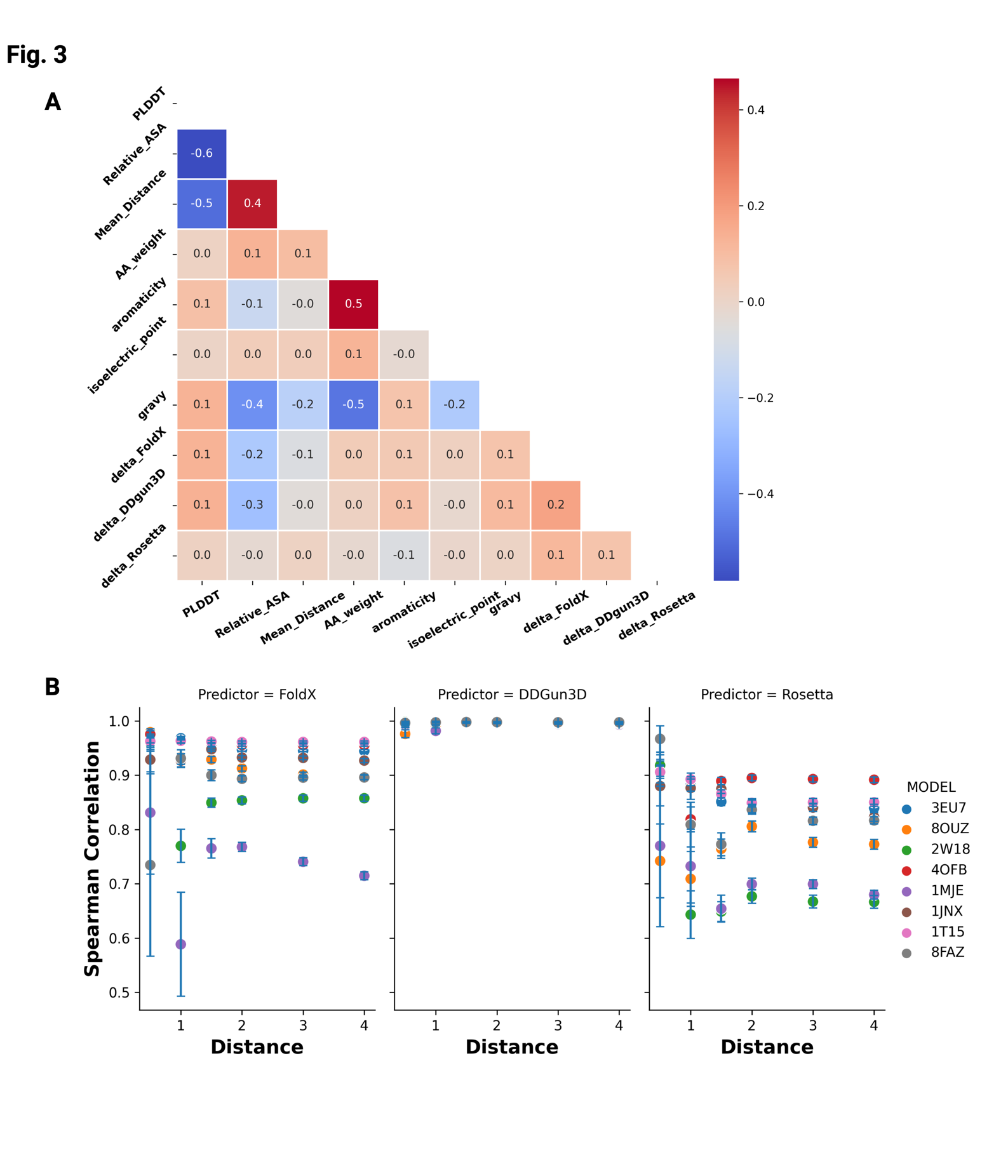


Figure 3: Monotonic association of difference between the ΔΔG from AF2 structures vs experimentally-derived structure as the wildtype template analyzed with FoldX, Rosetta and DDGun3D with the features extracted from AlpahFold2 structures and per residue distance between the superimposed AlphaFold2 structure onto the experimentally-derived structure.

A. heatmap of the spearman rank correlation coefficient denoting the monotonic association of the features derived from AlphaFold2 structures and deltas of the predicted ΔΔG derived from AlphaFold2 structures and experimentally-derived structures analyzed with FoldX, Rosetta and DDGun3D. B Scatterplot denoting the spearman rank correlation of the difference between the predicted ΔΔG from AlphaFold2 structures vs experimentally-derived structure as the wild-type template analyzed by FoldX, DDGun3D and Rosetta stratified by the per residue distance between the superimposed AlphaFold2 structure onto the experimentally-derived structure. The distance of the x-axis is limited to 4Å


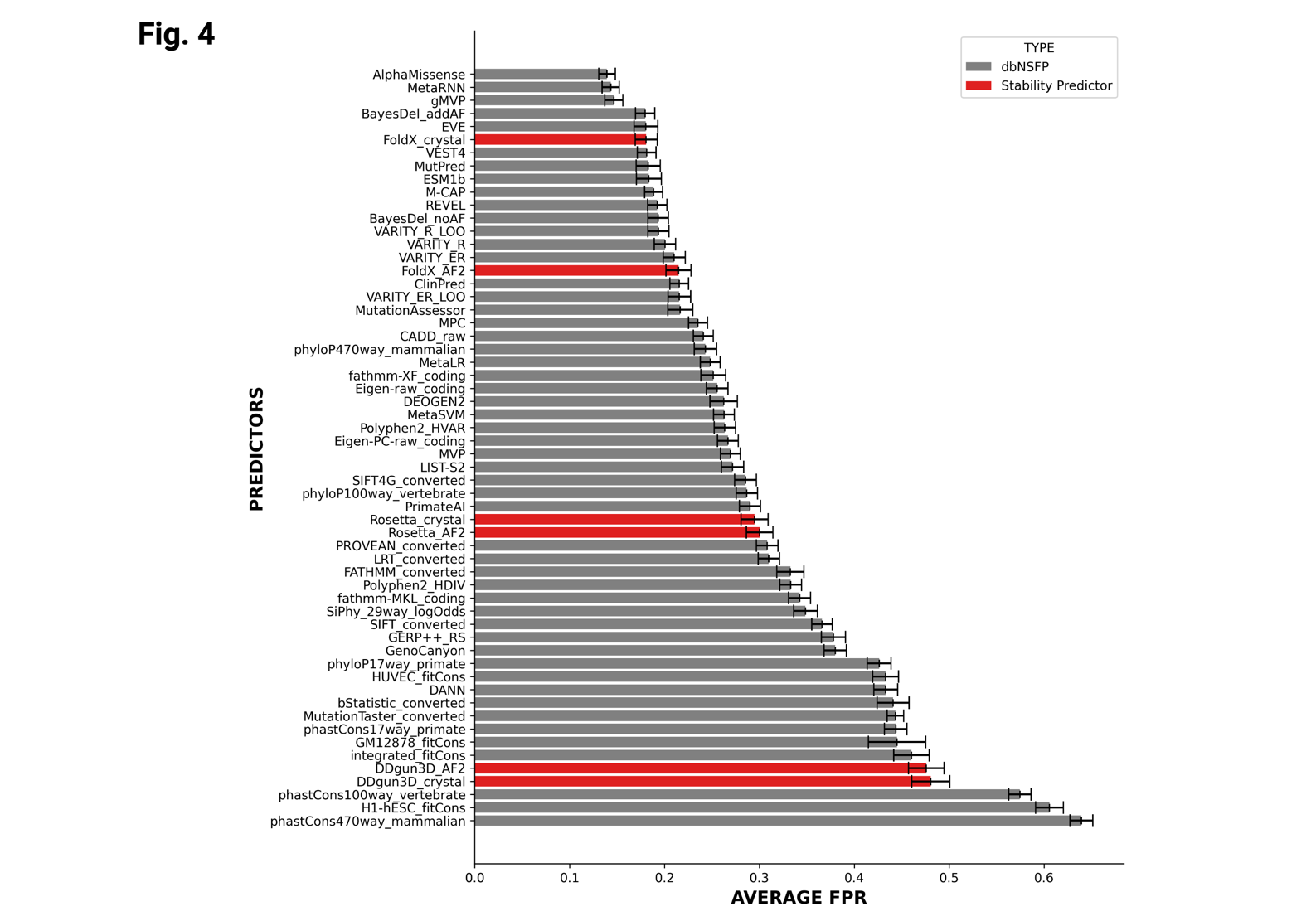


Figure 4: Barplot with 95% confidence intervals describing the average false positive rates for all dbNSFP Insilco missense predictors and stability predictors across the genes BRCA1, BRCA2, PALB2 and RAD512C
